# Visual Modeling Languages in Patient Pathways: Scoping Review

**DOI:** 10.2196/55865

**Published:** 2024-11-15

**Authors:** Binyam Bogale, Märt Vesinurm, Paul Lillrank, Elisabeth Gulowsen Celius, Ragnhild Halvorsrud

**Affiliations:** 1 Department of Neurology Institute of Clinical Medicine University of Oslo Oslo Norway; 2 Institute of Healthcare Engineering and Management, Department of Industrial Engineering and Management Aalto University School of Science Espoo Finland; 3 Department of Neurology, Oslo University Hospital and Institute of Clinical Medicine University of Oslo Oslo Norway; 4 Sustainable Communication Technologies SINTEF Digital OSLO Norway

**Keywords:** patient pathways, visual modeling languages, business process model and notation, BPMN, unified modeling language, UML, domain-specific modeling languages, scoping review

## Abstract

**Background:**

Patient pathways (PPs) are presented as a *panacea solution* to enhance health system functions. It is a complex concept that needs to be described and communicated well. Modeling plays a crucial role in promoting communication, fostering a shared understanding, and streamlining processes. Only a few existing systematic reviews have focused on modeling methods and standardized modeling languages. There remains a gap in consolidated knowledge regarding the use of diverse visual modeling languages.

**Objective:**

This scoping review aimed to compile visual modeling languages used to represent PPs, including the justifications and the context in which a modeling language was adopted, adapted, combined, or developed.

**Methods:**

After initial experimentation with the keywords used to describe the concepts of PPs and visual modeling languages, we developed a search strategy that was further refined and customized to the major databases identified as topically relevant. In addition, we consulted gray literature and conducted hand searches of the referenced articles. Two reviewers independently screened the articles in 2 stages using preset inclusion criteria, and a third reviewer voted on the discordance. Data charting was done using an iteratively developed form in the Covidence software. Descriptive and thematic summaries were presented following rounds of discussion to produce the final report.

**Results:**

Of 1838 articles retrieved after deduplication, 22 satisfied our inclusion criteria. Clinical pathway is the most used phrase to represent the PP concept, and most papers discussed the concept without providing their operational definition. We categorized the visual modeling languages into five categories: (1) general purpose–modeling language (GPML) adopted without major extension or modification, (2) GPML used with formal extension recommendations, (3) combination of 2 or more modeling languages, (4) a developed domain-specific modeling language (DSML), and (5) ontological modeling languages. The justifications for adopting, adapting, combining, and developing visual modeling languages varied accordingly and ranged from versatility, expressiveness, tool support, and extensibility of a language to domain needs, integration, and simplification.

**Conclusions:**

Various visual modeling languages were used in PP modeling, each with varying levels of abstraction and granularity. The categorization we made could aid in a better understanding of the complex combination of PP and modeling languages. Standardized GPMLs were used with or without any modifications. The rationale to propose any modification to GPMLs evolved as more evidence was presented following requirement analyses to support domain constructs. DSMLs are infrequently used due to their resource-intensive development, often initiated at a project level. The justifications provided and the context where DSMLs were created are paramount. Future studies should assess the merits and demerits of using a visual modeling language to facilitate PP communications among stakeholders and use evaluation frameworks to identify, modify, or develop them, depending on the scope and goal of the modeling need.

## Introduction

### Background

The concept of patient pathways (PPs) has been widely used to improve health system functions across a range of health conditions, care levels, and regions [1]. PP implementation spans from emergency care [2] to specialized fields such as cancer care [3,4], with recent adaptations to address conditions like the COVID-19 pandemic [5]. Effectiveness reviews of PP implementation indicated improved patient outcomes, reduced length of stays and cost of care, enhanced teamwork, and improved documentation [6-11]. Depending on the type of pathology and nature of the organization where the concept was introduced, the evidence on the effect of the outcomes is inconclusive. The confusion around the concept [12], the variability in its quality [13], and the deficiencies in the process of contextualization were among the implicated factors in the effectiveness studies that reported on patient, health system, and finance outcomes [14]. Seys et al [14] indicated the dual complexity, that is, the PP itself is a complex concept implemented in a complex health system. This calls for clarity in all aspects, including simplification of the description to facilitate communication and common understanding to enhance its effectiveness [15-17].

Multifaceted factors pose varying challenges to maximizing the benefits of PPs. Yet, there is an ongoing discussion on the unified definition and frameworks for their development, implementation, and evaluation [12]. Efforts are being made to synthesize and consolidate the various terms used and their definitions since the first identification of several alternative names is in action [18]. Furthermore, 84 different definitions along with the differing focus of pathways in the United States and the United Kingdom were identified shortly after their use [19]. One of the pioneering definitions, also adopted by the European Pathway Association, states that “A care pathway is a complex intervention for the mutual decision-making and organization of care processes for a well-defined group of patients during a well-defined period” with characterizing features [20]. To identify studies for an effectiveness review, Kinsman et al [21] proposed an operational definition, later refined by Lawal et al [22], and validated it in an emergency medicine review [23]. The use of terms evolved from merely clinical orientation of care provision (clinical pathways) to the inclusion of more stakeholders and from elements of care organization (care pathways) to the inclusion of multiple levels of care (integrated pathways or integrated care pathways) to develop a patient-focused systemic approach (PPs). Schrijvers et al [24] argued that adding qualifiers such as in “integrated care pathway” is unnecessary because the care pathways are integrated by definition. Such comments seem not to have held on because the most recent concept analysis paper proposed an even longer term, a “patient-centered care pathway,” showing an increase introduction of terminologies [12]. Their proposed definition, “a long-term and complex managerial intervention adopting a systemic approach, for a well-defined group of patients who journey across the entire continuum of care, from prevention and screening to recovery or palliative care,” with several attributes [12], however, is indicative of perspectives added toward comprehensive and patient-centric concepts. The key characteristics and elements were listed, and their importance was stressed beyond the proposed definitions in each article [12,19,21,22], with slight variations. In this review, we use “patient pathways” to represent the concept from a patient-centered care perspective [12] while using the criteria proposed for a Cochrane systematic review [21,22] to identify the articles. Patient journey studies, which focus on patient-centric mapping and analysis of health care delivery processes, are increasingly being introduced to the scene [25]. To simplify, we use “patient pathways” to represent a plan as a blueprint of the care process, while patient journey denotes an individual experience of the planned PPs revealed retrospectively. In this review, we used different terms interchangeably in the identification and review of articles. Confusion in the definition and conceptualization leads to variabilities in the analysis and modeling of PPs [12].

The rampant siloed and local productions with varying representations of a PP [1] call for more standardized ways to describe and communicate PP to have a shared understanding of the concept. This can be argued in the same manner as consensus frameworks have been proposed [12,14,26,27] in an attempt to standardize the stepwise development, implementation, and evaluation of PPs. The standardization process starts with the modeling languages that are used to describe and communicate PPs. Particularly, visual modeling “not only enables easy interpretation but moreover denotes a useful means for communication and understanding” [28] of a process. Graph-based formalism is one of the two most common process-modeling approaches [29]. The benefits of visual modeling over other forms of representation have been extensively discussed in business process modeling, although there remains a scarcity, particularly in the PP domains, of effectiveness studies using a traditional approach [29-31]. Lack of a common language also exacerbates the interoperability challenge in the increasingly digitized care processes, including digitized PPs integration to other electronic patient records and quality improvement digital tools [32,33].

### Prior Systematic Reviews on Modeling Languages

The modeling of PPs can be done to understand and analyze the current state, which is often referred to as an “As-Is” model, or a redesigned or improved PP modeling can be done in a “To-Be” model. The “As-Is” model is often “data-driven” modeling that uses clinical data from various electronic tools, for example, electronic health records (EHRs) and registers. Clinical data-based mathematical modeling often focuses on exploring the patient journey retrospectively, the focus being on simulation studies, data mining, machine learning, and artificial intelligence to predict the most efficient path for a care process [33,34]. The “To-Be” model considers PPs as a complex concept, which is prescriptive in nature and created by a multidisciplinary team. One of the challenges for this model is the absence of a common process-modeling language that is complex enough to incorporate all the necessary aspects in a model while being simple enough to be used by nonmodeling domain experts, either manually or digitally [35].

There are many different process-modeling languages coming from different scientific traditions, and their characteristics and intended use vary greatly [36-38]. However, the complex nature of health care delivery has led to a modest uptake of business process–modeling languages that are shown to be useful in sectors with predictable work processes [39]. Although choosing a modeling language has generally been a challenge [37,38], there are a few systematic reviews that have targeted specific modeling languages or notations in the care process [39-41]. Specific to PP-related concepts, there is a comprehensive review on modeling [42] while other reviews are more inclined toward data-driven pathway description and optimization [36]. The most similar review to our scoping review was conducted by Mincarone et al [37] in which only standardized modeling languages were included on the entirety of care process, which is wider in scope than PPs. To improve the domain expressiveness of a modeling language, extending or combining a general purpose–modeling language (GPML) or developing a domain-specific modeling language (DSML) can often be done. To our knowledge, the justification for and the “how” of such efforts have not previously been summarized. In addition, focusing on standardized languages and notations potentially excludes DSMLs, which are costly to develop but may display higher expressiveness than the GPMLs, including their domain-extended versions.

### Goal of This Scoping Review

**Figure 1:**
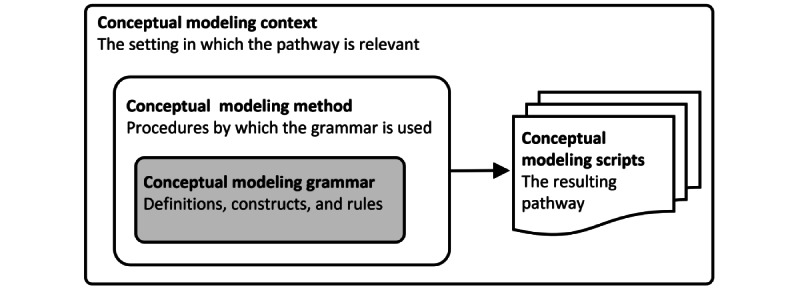
Framework for research on conceptual modeling (adapted from Wand and Weber [44]).

This review’s scope is to include both DSMLs and standardized GPMLs with emphasis on how they were used to meet the domain-specific requirements. Our focus is on visual modeling languages but not on 1D textual languages as delineated by Moody [43]. Visual languages in this case include ontological modeling languages that depict terms and concepts with their relationships in a visual manner. Most of the PPs are described in natural languages, with flowcharts and tables accompanying for simplicity, but we did not include simple flowcharts and diagrams as a visual notation. Wand and Weber [44] introduced a framework for research on conceptual modeling consisting of 4 elements to ease communication. To reduce potential confusion around terms in this scoping review, we introduce an adapted version of this model (Figure 1, adapted from Wand and Weber [44]). The modeling *grammar* constitutes the inner core because it provides definitions, constructs, and rules to produce a model. The modeling *script* is the end product of the modeling process. The modeling *method* describes how grammar can be used to produce a script. The modeling *context* has a wider perspective and describes the setting in which the modeling occurs. We do not intend to provide a comprehensive review of the *script* because our focus lies at the core of the framework. While grammar is the core, modeling method and context are of interest in this review because this review aims to contribute to the different ways in which a modeling language is presented.

### Review Questions

A scoping review was conducted to systematically identify and map visual modeling languages used to describe and communicate PPs. The review addresses the following review questions:

- Review question 1: Which visual modeling languages were used in the modeling of PPs?
- Review question 2: What are the justifications provided to adopt, adapt, or develop a visual modeling language to describe and communicate PPs?
- Review question 3: Within what contexts are the visual modeling languages applied in PP projects?

## Methods

### Overview

The reporting of this review follows the systematic review extension for scoping reviews PRISMA-ScR (Preferred Reporting Items for Systematic Reviews and Meta-Analyses Extension for Scoping Reviews) [45]. We followed the methodological framework proposed by Arksey and O’Malley [46], which was further improved by Levac et al [47] and the Joanna Briggs Institute [48], to conduct this scoping review. The review questions were proposed and refined after a thorough exploration of the existing synthesis on the topic, as presented earlier. The rest of the framework is integrated into the reporting format as presented in subsequent sections.

### Eligibility Criteria

To be included in the review, papers needed to satisfy the operational definitions (Textbox 1) for both PP and a visual modeling language. The article’s main aim could be either a theoretical discussion or empirical research on a modeling language that is created, adopted, adapted, or combined. Peer-reviewed journals, conference and congress proceedings, and gray literature that are accessible on the internet, regardless of the geographical location, type of health condition, and publication date up until the last systematic search dated November 21, 2022, were eligible. For papers published in a conference proceeding, we searched for and opted to include the more complete peer-reviewed publication in a scientific journal, if it existed, in expectations of detailed information. Only papers published in English were considered. No restrictions on study design, population characteristics, type of health care facility, or level of care were applied.

Papers were excluded if their focus was mainly on mathematical models, simulation studies, or machine learning without having information to report on the visual graphical presentation of a model for facilitating communications, including to nonmodeling expert stakeholders. Comprehensive reviews [35,36,42] have been done to cover modeling methods and languages applied in data-driven retrospective models. According to the conceptual framework proposed by Wand and Weber [44], we excluded papers that have primarily emphasized the presentation of a pathway using a known modeling language without an adequate description of how it is being used differently in the domain it is being applied to. Papers uniquely reporting modeling tools (software artifacts) without discussing the underlying modeling language used, or its semantics and ontologies for reasons of extension or visual presentation were excluded. Ontology-based and semantic modeling languages, with or without the inclusion of visual modeling as an output, were included in the review given the aim of this study is to facilitate communication of PPs among the stakeholders. According to a review by Zarour et al [49], representation of business process model and notation (BPMN) extensions can be of one of the 3 formats: metamodel, XML Schema, or graphical elements, and therefore, we included metamodels in this review.

We excluded simple flowchart presentation of pathways, which is the most common description and communication approach in medical domain but is now headed toward extinction [42] because the more mature notations were extensions of it and the relevance of including and discussing its use was perceived to be not adding to the standardization and wide acceptance of visual modeling for PP discussion. The enforcement of inclusion criteria began with the development of search strategies broadly and gradually narrowed down during the selection process. In the initial screening stage, articles were included if they mentioned or described 2 concepts, PPs and visual modeling language, in the abstract. The second stage involved a closer examination of these concepts. For instance, papers mentioning terms related to PPs but discussing broader concepts such as general care processes were excluded. Similarly, articles mentioning a visual modeling language but failing to describe how it was used in their research context were also excluded.

**Components and Definitions according to population, concept, and context framework proposed by the Joanna Briggs Institute.**

**Population and Participants**

- Regardless of the professional background of the experts behind the modeling language; irrespective of the health condition and level of care. The paper can be a theoretical exploration of how existing process-modeling languages, particularly general purpose–modeling languages (GPMLs) applied to other domains, can be adopted or adapted. This extends to the development of domain-specific modeling languages (DSMLs) for describing and communicating patient pathways (PPs) to facilitate common understanding among intended stakeholders.

**Concept**

Two concepts are as follows:

- Patient pathways: We based the PPs concept definition on a refined operationalization criterium by Kinsman et al [21] and by Lawal et al [22] in that they used it to identify articles for a Cochrane systematic review. Articles primarily focusing on theoretical discussions of a modeling language without empirical studies (including a case study for the sake of demonstration) might not reflect the aforementioned criteria. Such articles were included as long as the authors clearly stated that the application is for patient pathway concepts. As the terms used have evolved through time and are sometimes used interchangeably, we relied on the concept definition and explanations by the authors. Papers containing terms and phrases that are often used interchangeably, such as care process, workflow, etc were excluded if the paper did not explicitly state the PP concept dealing both with the organization and clinical part of the care.
- Visual modeling language: general-purpose or domain-specific modeling languages that may or may not be standardized, can be graphical, rule-based, or combined presentations, aimed at describing a PP, regardless of the origin and the extent to which a given modeling language has been implemented. The modeling language can be adopted, adapted, combined (as in complementary combinations of stand-alone modeling languages), or developed to model a PP and can or cannot have digital applications and tools accompanying the language. Modeling languages used in mathematical models, simulations, artificial intelligence, and machine learning are outside the scope of this review

**Context**

- The modeling language applied regardless of disease or health condition, treatment and intervention options, clinical settings, and service delivery level (primary, secondary, or tertiary care facilities). No restrictions based on geographical location and scope within the location.

### Information Sources

We conducted searches in the following databases: MEDLINE via PubMed, PsycINFO, Embase, CINAHL, and Scopus after iteratively developing search strategies. We used Joanna Briggs Institute [48] population, concept, and context framework to exhaustively list search terms under each component. The following initial search terms were used: “Patient Pathway*,” “Care Pathway*, “Clinical pathway*,” along with the Medical Subject Heading term “Critical pathway/” in MEDLINE and CINAHL databases to decide on what additional terms to combine with them to identify papers relevant to our inclusion criteria. For words describing modeling language and related concepts, terms like “language*,” “model*,” “framework*,” “formalism*” were used and search was expanded by including additional similar terms from retrieved articles. We narrowed the scope of the terms by adding descriptors. The search strategies were drafted in consultation with an experienced librarian and customized to each database after iterative rounds of improvements. We limited our search strategy to the title and authors’ keywords after discovering that including the abstract greatly increased the number of search hits. The final search strategies for each selected database are available in Multimedia Appendix 1. Gray literature search and hand searches of relevant articles referenced by included papers were done by the first author. After learning that the IEEE website has useful collections on the topic, we made searches in addition to the initial database search. As a forward search strategy, Web of Science and Google Scholar databases were used.

### Selection of Sources of Evidence

The librarian used EndNote (Clarivate) to deduplicate the retrieved articles. After importing them into Covidence (Veritas Health Innovation Ltd) software, any remaining duplicates were identified and automatically removed using the software’s built-in feature. In total, 2 reviewers independently screened publications in 2 stages after importing all the retrieved papers into the Covidence software [50]. To have consistency in the screening process, we defined and discussed the inclusion and exclusion criteria and documented them in the Covidence software for easy referencing and to use the automated word detection features of the software. Accordingly, the 2 reviewers conducted the screening of the title, index words, and abstract at stage 1 and full-text review screening at stage 2 guided by the predetermined inclusion and exclusion criteria. In case of discordance, a third reviewer voted the article as in or out in stage 1 screening, and a consensus-based resolution of disagreements was reached in stage 2 between the 2 reviewers.

### Data-Charting Process

A data-charting form was developed by the first author and discussed with the second reviewer who participated in all the screening stages. The 2 reviewers discussed and iteratively improved the data-charting form while retrieving the results from the included papers. The data-charting form was developed to capture key information about the modeling language in addition to the identifying characteristics of the papers (author, title, date of publication, region or country, and journal or source). The form featured a free text section dedicated to capturing details about the modeling language including its name, descriptions, justification, and its application within the research context. In addition, categorical information, such as yes or no responses, was included accompanied by a follow-up free text area. For example, questions like “Is there an associated digital tool?” allowed for documentation of tool names, if applicable. Regarding the concept of PPs, we extracted the phrases used to define or represent the concept, as well as any stated definitions within the document. All the data-charting process was conducted using the Covidence software and eventually exported to a Microsoft Excel spreadsheet.

### Synthesis of Results

The synthesis of the results followed the review questions where we descriptively categorized the modeling languages into thematic groups. We also thematically analyzed the justification, purposes, and context in which the modeling language was created or implemented.

## Results

### Selected Articles

**Figure 2:**
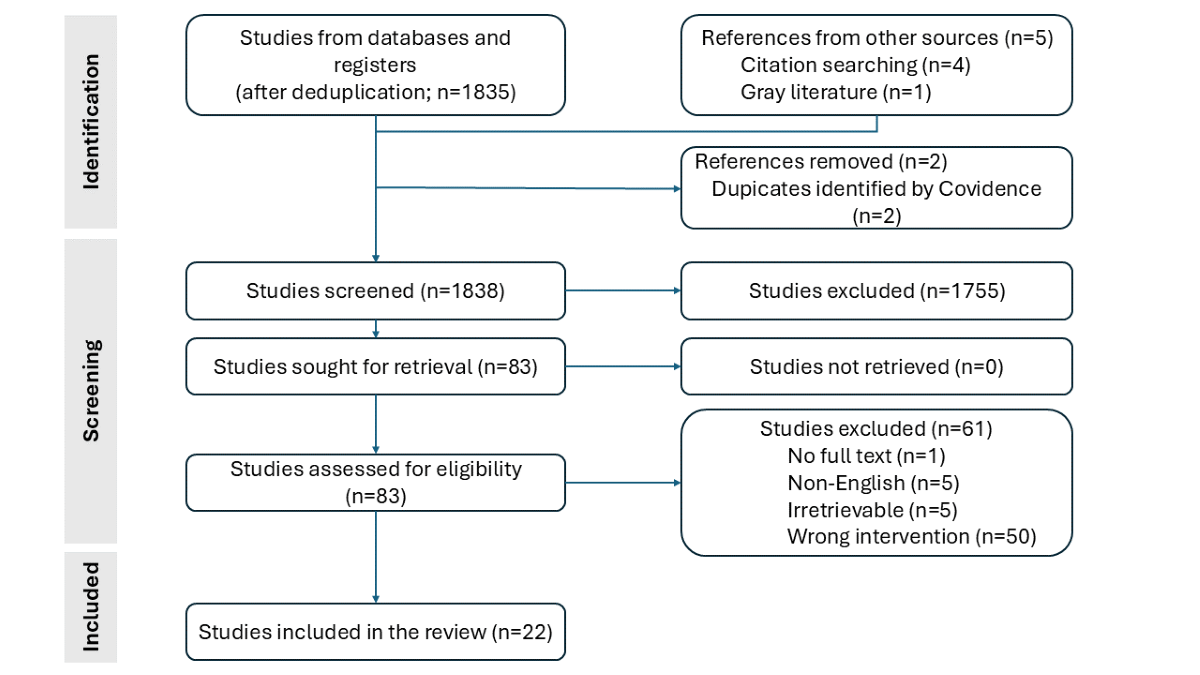
PRISMA (Preferred Reporting Items for Systematic Reviews and Meta-Analyses) flowchart.

We imported a total of 1835 articles from the selected databases to Covidence after removing duplicates (Figure 2). In addition, we discovered 5 more articles through hand searching, including gray literature searches from the Object Management Group (OMG). Of the 22 papers included in our review, half were published in conference proceedings and mainly retrieved from IEEE. The other half, except for 1 article from gray literature, were obtained from peer-reviewed scientific journals (Table 1). We included papers that were published as far back as 2008 to as recent as 2022. Most of the papers were from Europe (Germany and Italy), and almost all of them were from high-income countries. We found that more than 1 modeling language was contributed by similar groups of coauthors in another paper included in the review.

**Table 1:** Summary information of the included publications.

Study	Country	Publication source (categorized)	Modeling language used	Case study or demonstrated on
Scheuerlein et al [51], 2012	Germany	Journal	*BPMN*^a^ (*tBPM*^b^)	Colon and rectum carcinoma (treatment of)
Barbagallo et al [52], 2015	Italy	Journal	*BPMN*	Multiple surgical conditions
Ferrante et al [53], 2013	Italy	Journal	*UML* ^c^	(Post) stroke rehabilitation
Ferrante et al [54], 2016	Italy	Journal	*UML*	Stroke rehabilitation
Mauro et al [55], 2010	Germany	Proceeding^d^	*UML*	Not mentioned
Zerbato et al [56], 2015	Spain and United States	Proceeding	*BPMN for process and UML for data*	Catheter-related bloodstream infections
Braun et al [57], 2016	Germany	Proceeding	*BPMN4CP 2.0* ^e^	Stroke
Richter and Schlieter [58], 2019	Germany	Proceeding	*BPMN, with Quality BPMN, with quality indicator extension*	Integrated stroke care
Tehrani et al [59], 2012	United Kingdom	Proceeding	*BPMN, with norm extension*	Major gynecological surgery
Combi et al [60], 2017	Italy	Journal	*BPMN and DMN* ^f^	COPD^g^
Sooter et al [61], 2019	United States	Journal	*BPMN and DMN*	Contraceptive use
Object Management Group Healthcare Domain Taskforce [62], 2020	United States	Gray	*BPM+health* ^h^	Multiple conditions
Bowles et al [63], 2018	Germany	Proceeding	*BPMN and LES* ^i^	Diabetes and hypertension
Ardito et al [64], 2020	Italy	Journal	*Metamodel; EER* ^j^ *, BPMN and UML*	Headaches
Iglesias et al [65], 2022	Spain	Journal	*TP-VML* ^k^	Catheter-related bloodstream infection
Trajano et al [66], 2021	Brazil	Journal	*MedPath*	85 different care pathways: including low-back pain, diabetes, syphilis, etc
Burwitz et al [67], 2013	Germany	Proceeding	*CP-Mod*	Tooth extraction
Shitkova et al [68], 2015	Germany	Proceeding	*Icebricks*	Cardiac diseases
Li et al [69], 2008	China	Proceeding	*OWL* ^l^	None, theoretical
Ye et al [70], 2008	China	Proceeding	*CPO* ^m^ *, domain ontology, and SWRL* ^n^	None, theoretical
Nishimura et al [71], 2014	Japan	Proceeding	*CHARM* ^o^ *: ontological*	7 different diseases
Abidi and Abidi [72], 2012	Canada	Proceeding	*OWL*	Prostate cancer

^a^BPMN: business process model and notation.

^b^tBPM: tangible business process modeling.

^c^UML: unified modeling language.

^d^Includes congress, conference, symposium, and published papers mainly on the IEEE and IEEE Xplore websites.

^e^BPMN4CP 2.0: business process model and notation for clinical pathways 2.0.

^f^DMN: decision model and notation.

^g^COPD: chronic obstructive pulmonary disorder.

^h^BPM+Health: business process management for health care.

^i^LES: labeled event structure.

^j^EER: enhanced entity relationship.

^k^TP-VML: task planning visual modeling language.

^l^OWL: web ontology language.

^m^CPO: clinical pathway ontology.

^n^SWRL: semantic web ontology rule language.

^o^CHARM: convincing human action rationalized model.

### Modeling Languages

#### Overview

Modeling languages that have their origin and wider uptake outside of the health domain and others developed within health domains were identified. We have collectively called the former GPML, and the latter DSML. In this context, GPML means a widely used modeling language or notation to visualize processes that transcend the health domain. These modeling languages can be used in the health care domain either (1) directly and without major extension or formal combination, (2) by extending the specifications following extension recommendations, or (3) by combining to enhance their expressiveness. On the basis of the presentation of the output, some can be formal notations and others visually presented concept relationships. To provide a detailed and simplified account, we grouped the included modeling languages into categories mentioned in subsequent sections.

#### GPMLs: Without a Formal Extension to the Domain Requirement

This category contains adopted process-modeling languages that are standardized and widely applied in other sectors. The 2 OMG standards, BPMN [51,56] and unified modeling language (UML) [53-55], were used without any demonstrated extension or modification to the original specifications. The methodology to enhance GPML uptake, especially to include nonmodeling experts in the modeling process, was not considered as a change to the modeling grammar and syntax. In the case of tangible business process management (BPM) [51], the authors used physical icons instead of digital tools that enhanced the participation of the domain experts. Zerbato et al [56] stated extending with additional time primitives to represent temporal constraints, which is not supported by the BPMN version that they have used. However, the details of the extension and its outcome were not provided. In the latest of the 2 papers [54], Ferrante et al [53] discussed the method around the modeling process to the original paper where UML is used to model the stroke rehabilitation pathway.

#### GPMLs: With a Formal Extension to the Domain Requirement

By adapting the widely used standardized notation, Braun et al [57] provided an extension formalism called BPMN4CP (business process model and notation for care pathways) to include care pathway—specific aspects. The revised BPMN4CP 2.0 covers additional domain-specific requirements to the original recommendations. Whereas Richter and Schlieter [58] extended BPMN to add the quality indicator specifications based on the BPMN4CP extension framework. Tehrani et al [59] extended the BPMN specification from the results of their norm analysis, together with organizational semiotic methods, which focus on describing the human behavior and conditions under which the human behaviors occur. In the notation, the extension was indicated by labeling (N#). In all 3 cases, it is imperative to take advantage of the widely used process models with their extension possibilities.

#### A Combination of Modeling Languages

To satisfy the domain requirements and specific needs that arise in the modeling processes, 2 or more visual modeling languages were used in combination, often complementing one another as required by the nature of each part in a given model. The OMG BPM+health provides a possibility to combine 3 independent modeling languages (BPMN, case management model and notation [CMMN], and decision model and notation [DMN]) [62], while Combi et al [60] and Sooter et al [61] combined BPMN and DMN in their respective studies. Extensive use of other modeling languages in combination, including the use of data modeling to identify comorbidity was presented in 2 instances [63,64]. One could argue that these may not be identified as visual modeling languages.

#### Developed DSMLs

Three articles [65-67] discussed the development of a DSML; one was called MedPath, which was specifically developed for the PP modeling process [66], and the other 2 radically improved [65,67] the base modeling language that inspired the development of specific DSML to model PPs.

#### Ontological Modeling Languages

This category is made acknowledging the differences between the promises of visual modeling and ontologies. While visual models do not intend to present a complete description of the domain and the constructs, rules should reflect some ontological commitments. Ontological models go beyond such restriction and cover a possible set of concepts and premises in a domain [72]. Ontology-based modeling languages were supported by other methodological approaches, such as semantic and norm analyses, and specifically dealt with terms and expressions to satisfy the PP requirements. Li et al [69] built an ontology chart that is presented in a diagrammatic visualization of the constructs following semantic and norm analysis. The widely used web ontology language and Protégé (a free, open-source ontology editor and framework for building intelligent systems) were used to personalize the pathways. To align institution-specific PPs in an automated solution, Abidi and Abidi [72] used semantic web-based modeling using web ontology language to align pathway ontologies. In another study, semantic web ontology rule language was used to represent the temporal relationship that was not covered by the time subontology of the clinical pathway ontology together with domain ontology [70]. Aimed at confirming the practical ability of convincing human action rationalized model to represent medical actions, promoting knowledge sharing, and inheritance in a computer-interpretable way, Nishimura et al [71] built the convincing human action rationalized model tree that contributed to finding the commonalities, variations, and reasons for differences among pathways.

### Justification to Adopt, Adapt, Combine, or Develop Modeling Languages for PPs

The justification provided by authors for the identification, selection, use, and development of each modeling language varied across many different factors. These include but are not limited to the aim, level of analyses, the composition of the group behind the language, and when it was proposed during the progressive maturity of visual process languages.

#### Justification to Adopt a Visual Modeling Language

The justification given to adopt the GPMLs without extension was mainly to test the applicability of the modeling languages in health care domain and how the digitization process can be supported. The popularity and wider tool support made it easier to identify from the vast selection that already exists. Some authors provided comparative analysis to justify their choice.

#### Justification to Extend a Visual Modeling Language

To extend a modeling language, the main rationale emanates from the understanding of the deficiencies of GPMLs to meet the PP domain requirements. Apart from the popularity and resources around the modeling language, the presence of extension metamodel and the frameworks to guide the extension process are necessary to begin assessing whether the requirements can be met without extension (Table 2).

**Table 2:** The justification to extend a modeling language.

Study	Modeling language	Justification to adapt or extend
Braun et al [57], 2016	BPMN4CP^a^	Domain requirements are not fully represented, and the extension procedure needs to follow a framework
Richter and Schlieter [58], 2019	BPMN^b^, with quality indicator extension	Widely accepted and established standard Gives a metamodel, suitable for extension Presence of extension framework [56] to build on
Tehrani et al [59], 2013	BPMN, with norm extension	BPMN is “a rigorous method that provides a rich set of techniques and notations for process modeling” Absence of techniques to describe human behavior and the conditions under which the behavior occurs

^a^BPMN4CP: business process model and notation for clinical pathway.

^b^BPMN: business process model and notation.

#### Justification to Combine Visual Modeling Languages

No single modeling language can adequately cover the requirements because the health care domain in general, and the PP modeling in particular, is a complex process. In this category (Table 3), the main rationale for combining modeling languages emerges from the conviction that complementing the deficiency of one model with the strength of the other is possible. In three of the cases, all the combinations were made progressively and included the specifications from OMG [60-62].

The health care Domain taskforce of the OMG has introduced a field guide called *shareable clinical pathways version 2.0* introducing amalgamation of their 3 standard notations for clinical pathway modeling [62]. Although not officially endorsed as a standard, it serves as a valuable discussion paper. The primary objective is to establish a modeling technique that is universally comprehensible among various stakeholders, including business analysts, health care professionals, and IT developers.

Implementing health information technology based on a care model that is universally developed and understood contributes to the efficiency, cost-effectiveness, and quality of health care delivery. The guide proposes the use of BPMN for prescribed models, CMMN as a complement for actual workflows, and DMN to address decision modeling, encompassing a complex set of factors to arrive at the most appropriate clinical decision. The suggested combination facilitates the creation of business flow diagrams and decision tables, offering coverage for clinical, administrative, and revenue cycle processes. This model, using a blend of these languages, addresses diverse aspects essential in the health care domain and is standardized for seamless sharing across organizations.

The BPM+ community of interest was assigned the task of examining the alignment between 2 OMG standard notations, namely BPMN and DMN. The focus was on bridging the gap between narrative guidelines and their digital representation with the aim of achieving a “true integration” of guidelines and pathways within electronic medical record systems. Sooter et al [61] illustrated the feasibility of modeling a clinical guideline for contraception in a standardized format suitable for digitization. Their approach involved listing and defining all data points using a spreadsheet, which subsequently indicated an area for extension to the BPMN 2.0 specification. The team showcased the challenges encountered in mapping SNOMED-CT (Systematized Nomenclature of Medicine–Clinical Terms) codes and presenting all terms in a summary chart. The decision logic and the business process aspects related to contraceptive choices and delivery were effectively modeled with a low level of abstraction using a modeling tool.

Combi et al [60] developed a framework using DMN to model decision-intensive care aligned with clinical practice guidelines directed at clinicians. Simultaneously, they used BPMN for modeling the care organization aspect. The authors rationalized the misuse of BPMN for modeling the entire pathway, including clinical decisions, to conduct their research. However, they acknowledged the limitations of BPMN, particularly its shortcomings in fully supporting temporal relationships, domain knowledge, and the integration of complex structural data. The discussion explored the potential use of extensions to address these drawbacks. The steps outlined in the framework are more suitable for stakeholders with modeling expertise because the implementation of the modeling tool demands such skills.

Ardito et al [64] argued that finding a modeling language that is detailed enough to balance a machine-executable model yet simple enough to be human understandable is a challenging task. They proposed a modular approach for executing complex machine-level processes separately that uses a task-oriented chatbot approach based on the modeled pathways while interacting with the social media chatbot with the end user. The process used a stepwise approach and is method-intensive by nature, and it also includes patients, who are often forgotten as stakeholders in modeling efforts.

**Table 3:** The justification to combine modeling languages.

Study	Modeling languages	Justification to combine
Combi et al [60], 2017	BPMN^a^ and DMN^b^	Merely using a BPMN to model a decision-intensive care pathway is a misuse of the specification Need for a framework that guides the use of DMN for a decision and BPMN for a structured process
Sooter et al [61], 2019	BPMN and DMN	A “true integration” with electronic health records is not yet achieved The compatibility of the 2 modeling languages to change narrative guidelines to digital instantiations is not checked
Object Management Group Healthcare Domain Taskforce [62], 2019	BPM+Health^c^ (BPMN, CMMN^d^, and DMN)	Health care domain needs process models for prescriptive workflows, case models for reactive workflows, and decision models for complex business rules, hence appropriate modeling languages Need to use accepted standards to make pathways shareable
Bowles et al [63], 2017	BPMN and LES^e^	Other available presentations of CPsf for multimorbidity have inherent ambiguity. There is a need to resolve pathway conflicts using standardized, coordinated back-end and front-end models
Ardito et al [64], 2020	Metamodel; EER^g^, BPMN, and UML^h^	Finding a balance between modeling language expressiveness and the automated execution of modeled processes is difficult, and investing in it is unprofitable. To find the balance that does not require the domain experts to adopt a complex process-modeling language, what about dedicating the burden to an executing module—a chatbot engine?

^a^BPMN: business process–modeling notation.

^b^DMN: decision model and notation.

^c^BPM+Health: business process management for health care.

^d^CMMN: case management model and notation.

^e^LES: labeled event structure.

^f^CP: clinical pathway.

^g^EER: enhanced entity relationship.

^h^UML: unified modeling language.

#### Justification to Develop a DSML

Justifications to develop a modeling language included verbosity and issues related to customization and execution, limitations to cover PP domain requirements sufficiently, and needs such as integration. Furthermore, using domain-oriented existing languages was mentioned in this context (Table 4).

MedPath [66] is a process-based DSML that was developed to capture all the components in a clinical context while minimizing the verbosity challenges encountered by adopting the GPMLs. The language has syntax, semantics, and a visual notation. MedPath is a layer between the expert who develops a model and the engine that translates the metamodel into visual elements to be easily understood by the domain experts. The authors stated that the language is designed to be comprehensive enough to capture all aspects of care organization, simple enough to be understood by health care domain experts and detailed enough to be integrated into the health information system.

The *openEHR* foundation strives for interoperable EHR systems and the use of standardized models in care processes and is also behind the task planning (TP) initiative. TP is a clinically oriented specification that also has a visual modeling language called task planning visual modeling language (TP-VML) [65]. The authors credit the BPMN extensions that are aimed at solving the temporal constraints to model clinical workflow. However, TP formalism can better represent the health care domain because it has a more domain-specific orientation than the extensions. The original TP-VML icons and semantics are not discussed, while the displayed visual notation in the paper references its source to the tool that the authors used. Possible extensions of the TP specifications were presented after a thorough analysis following the stepwise methods. The authors concluded that following the extension procedures suggested for BPMN to pathways by Braun et al [57], TP can be extended to include domain requirements and can be used for complex PPs as demonstrated in the case study on the catheter-related bloodstream infection. Future work should focus on furthering the decision logic specification to evoke rules from TP supported by an expression language and a basic metamodel of openEHR.

Burwitz et al [67] developed a DSML named CP-Mod based on the clinical algorithms basic process-modeling concepts and extended to include evidence-based medicine and decision support, classification of treatment alternatives, and time events and waiting periods following the requirement they set for PP modeling. The justification for using the base modeling concept and the need for extension in comparison to the widely used modeling languages were presented, and good arguments were made.

Icebricks [68] is a modeling methodology and digital tool that also provides a human-readable representation of care pathways. It meets the need for a notation that depicts activities, annotates information, and supports standardization while facilitating collaborative work with easy learning. The model can be exported to Microsoft Word with full process documentation, including diagrams (although not shown in the paper) and annotated information. The authors raised important questions that qualify a useful notation, but more emphasis was given to the methodology and the tool than to the core of the language.

**Table 4:** The justification to develop a modeling language.

Study	Modeling language	Justification to develop
Iglesias et al [65], 2022	TP-VML^a^	More clinically oriented business process management standards can provide an adequate representation of the temporal orientation of clinical workflow than BPMNb or its extension.
Trajano et al [66], 2021	MedPath	GPMLsc verbosity and not easily customized to medical context. Also sometimes lack infrastructure for integration and execution.
Burwitz et al [67], 2013	CP-Mod	Reviewed common modeling languages used against their set requirement and identified deficits. Clinical algorithm can be used as a base and organizational aspect and individual data can be added.
Shitkova et al [68], 2015	Icebricks	Generic modeling languages did not sufficiently cover PPd requirements. A notation needs to allow the representation of activities and process flows, annotate relevant information, and represent knowledge on an appropriate level of abstraction.

^a^TP-VML: task planning visual modeling language.

^b^BPMN: business process model and notation.

^c^GPML: general-purpose modeling language.

^d^PP: patient pathway.

### The Contexts in Which the Languages Were Applied

The context in which the modeling process occurred can take several aspects. Here, we focus on the profile of involved experts, the organizational structure, the coverage and scope, the tool support, the nature of the study, and the level of standardization, among other relevant information to a language. Additional discussion of the context is provided in Multimedia Appendix 2 [57-68] and for selected papers in the earlier section.

Almost all articles were method-intensive and primarily focused on theoretical discussion with empirical demonstration on selected medical conditions. Only 3 articles discussed the topic without demonstrating on a case [55,69,70]. While the majority demonstrated on a single condition, a few attempted the application on several conditions [52,63,66,68,71]. The field guide [62] and contraceptive guideline modeling [61] are meant to be applied widely at several levels of abstraction. One paper focused on interinstitutional care standardization [72], integration of PPs to deal with comorbidity [63], and that it can be applied at any level of abstraction.

The drivers of the modeling language creation or use are mostly modeling experts who attempt to involve novice modelers. The domain experts were also involved at some level in the presented concept or script ranging from design to the evaluation phases. Different techniques, such as making the vocabularies tangible [51] and producing a chatbot that facilitated the participation of novice modelers [64], were identified. The intention to simplify the modeling process for stakeholders with less modeling expertise was indicated by several authors. The use of modeling languages was tied to digitization in several of the cases, particularly to facilitate machine executability [52,64-66,68].

The OMG is responsible for UML, BPMN, as well as CMMN and DMN proposed for combination with BPMN [51-56,60-62]. Languages like TP-VML that have domain-specific engagement are linked to a foundation with a potential to be widely accepted [65].

Tool support is one of the main criteria for increasing the adoption and subsequent improvement of modeling languages. More than two-thirds of the modeling languages were accompanied by a tool or software artifact [52,54,55,61-67,69-71] and the remaining did not specify in the report.

### PPs: Terminologies and Concept Definition

The terms and operationalization of the concept synonymous with PPs varied among the included articles. The most frequently used phrase was “clinical pathways” alone or interchangeably with other phrases including “care pathways” or “integrated care pathways.” There were no justifications provided as to why a given terminology was adopted. The phrase “patient pathways” appeared in only 1 paper [58]. In most of the papers, the concept of PPs (or their synonyms) was briefly discussed in the Introduction section. Almost all referenced the definitions forwarded by de Bleser et al [19], Vanhaecht [20], or Kinsman et al [21], among others. Almost all included papers lacked operationalization of the concept in relation to the context.

## Discussion

### Principal Findings

We identified and categorized visual modeling languages used in the representation of PPs. In addition to the direct adoption of standardized and widely used modeling languages originating outside of the health domain, there are extensions and combinations of languages proposed to meet the domain requirements. We identified DSMLs that are also important contributions to the discussion of PP modeling efforts. The justifications for selecting a visual modeling language varied depending on the modeling scope and goal. The rationale to propose any modification to the language evolved as more evidence was presented following requirement analyses to support domain constructs. The direct use of standardized modeling languages without any domain-specific adaptation was done mostly to test whether the standards can also be applied to the health domain. After having evidence regarding the deficiencies to fully represent the domain needs, extension by addition and combinations of more than 1 standardized visual modeling language were introduced. The presence of extension formalisms for widely used standard modeling languages, specifically BPMN, is promising, while the process of standardizing those extensions remains unclear. Standing on the shoulders of previous extensions would contribute to subsequently expanding the specification to the domain requirements with minimum effort. There are valid reasons put forward by the DSML developers, but these need further discussion considering the cost, rate of uptake, and likelihood of standardization. Given that there are more mature languages with already advanced tools, including the advantage of execution languages to automate the model, it is essential to conduct an exhaustive comparison of which languages to choose or whether there is a need to develop a new one before embarking on the long journey.

The taxonomy that we created corresponds to the approaches used to find the best possible ways to appropriately model PP. Similar approaches were used in other related reviews mainly to facilitate the ease of understanding of such a complex field [12,42]. The purpose of using a visual modeling language, otherwise stated as “the dependent variable/the design goal” by Moody [43], was reflected in the included papers in various ways. It has been used to facilitate communication between designers and domain experts [51,66], involve patients to interact with their PPs [68], facilitate the digitization of the PPs [61-67], and facilitate integration with the EHR system [61]. The reviewed papers attempted to fulfill these purposes fully or partly.

The application of standardized modeling languages in the health care domain has previously been reported in systematic reviews [38,39]. This scoping review adds *the process* in which the GPMLs were being used to model PP-specific requirements. In this review, BPMN is categorized into GPMLs because its syntax can be used in various domains despite its business process–specific nature [56]. We believe that it is a good example to illustrate how GPMLs were being used to model PPs. From early experimentations to check for its suitability to model PPs [51,52,56], followed by the identification of domain-specific requirements resulting in extension approaches [57-59], to the recent recommendation to combine other specifications to overcome its inherent limitation [60-62], BPMN presented itself as a leading standardized process-modeling language for PP. Gartner et al [12] also reported in their review that the process nature is one of the most common attributes of care pathways and is modeled using BPMN or improved by combining with decision support modeling languages. The popularity, expressiveness and extensibility, and tool support with an execution language were presented as justifications for its use, which are parts of the parameters in most evaluation criterion frameworks [28,44,73]. According to a comprehensive systematic review, the relative simplicity of use with strong research and implementation experiences in nonhealth domains supported by OMG is convincing to agree to the notion that one should justify why they did not use BPMN over their chosen modeling language [74]. However, to fully represent the domain requirements, combinations of standards that cover aspects other than predefined and stable processes must be considered [62].

It is widely accepted that the use of DSMLs may boost the modeling practice and enhance the flexibility, maintainability, and sustained use of a model compared with using a GPML [74,75]. Developing a DSML is very costly and time consuming. It is premature to declare the impact and possibility of continued use of all the included DSMLs in this review because of their recent appearances [65,66]. However, the justifications for embarking on such a time-consuming and costly process need to be recognized to gain inputs to build the most expressive and simplified modeling language for the domain. For example, TP-VML aimed at replacing all the extensions to GPMLs that are not built to represent all the needs in the domain [65].

On the basis of the anatomy of visual modeling languages [43], a visual vocabulary (graphical symbols), grammar (a set of compositional rules), semantics (definitions of the meaning of each symbol), and its visual (concrete) syntax are included. Most articles included in this review did not specifically aim at the core aspects of a modeling language by discussing its grammar, ontologies, and semantics fully. As this scoping review aims at mapping but not quality appraising the modeling languages, we did not seek out further information for those developed or traced back to the original specifications for those adopted, adapted, or combined known visual modeling languages. There are frameworks with comprehensive evaluation criteria to measure the quality of a modeling language, although the criteria confuse the script with grammar [44]. A few papers included in this review also presented their criteria for selecting one modeling language over another. Future studies need to find a good framework and evaluate all modeling languages used in PP in general and DSMLs in particular. As clearly recommended by Gemino and Wand [73], a comparison of modeling languages should be based on their grammar (constructs and rules) rather than the scripts (specific models and end product of a modeling process).

The modeling and selection of the appropriate modeling language go in line with the data sources. Aspland et al [42] stated that there are 2 common ways of obtaining data: either data-driven approaches or through collaboration with those who interact with the pathway. They recommended coordinating both sources to advance on the advantages of each. The increasing presence of digital technology presents the opportunity to use a data-driven approach, which also gives the opportunity to evaluate progress in a dynamic manner [12,33,40]. More emphasis is being placed on the presence of an EHR to have access to reliable data at all phases to “identify the relationships between the context, the mechanisms, and the results obtained” [12]. Of note is the extensive use of other modeling languages that are more relevant to the modeling methods categorized as stochastic, data mining and machine learning, simulation, and optimization and heuristics by Aspland et al [42]. Our review encompasses the use of modeling languages for pathways created by multidisciplinary teams in a prospective manner.

With the increasing use of patient-centric pathway development [12], modeling languages that reflect the patient perspectives are of interest. The patient journey concept, or customer and user journey in a wider context, is getting more attention; although, the literature on the subject is incoherent [76]. One such language that covers the perspective of patients is the customer journey modeling language (CJML), which is making its way to the health domain [77] targeting novice modelers [78]. CJML is developed from the human end user’s perspective, as opposed to the software-centric UML and the business-centric BPMN (refer to the diagrams of CJML from one of the authors’ previous works for illustration purposes in Multimedia Appendix 3). This modeling language has been explored to support the design, management, and analysis of patient journeys. CJML is designed to capture both planned (prescribed) and actual patient journeys, allowing for the analysis of deviations and the inclusion of the end-users' experience [79]. CJML provides vocabulary, a metamodel, and a visual notation. It is easy to learn and does not require complex technical competence to use. CJML provides a graphical notation for the planned and actual journeys with specific constructs such as journey phases, experience, channel, and actor information, which is limited or lacking in other process-modeling languages [77-79].

### Limitations of This Scoping Review

We restricted our inclusion criteria based on several factors. Although this has merits to focus this review on modeling languages that emphasized description and communication of PPs, our narrow focus, including restrictions of the search strategies to the title and keywords only, might have excluded papers that have relevance regarding how the modeling languages were used to address domain issues while used in the modeling of data-driven pathways. Our effort to include DSMLs, which are not yet standardized, posed a challenge as to where to draw the line in the abundance of modeling languages [38,74] with varying levels of abstraction and granularity. We excluded simple flowchart and diagram presentations, the most common visual notations, because the scope of this review includes advanced languages. However, a few matured visual presentations with less structured description of the language might have been excluded. We did not include all the standards and accompanying modeling languages and methods that focus on standardizing interoperability between different IT systems, such as HL7 (Health Level Seven International), and textual languages that support international or interinstitutional coding to classify and standardize terminologies, such as SNOMED-CT [80]. We excluded the so-called task-based modeling languages commonly used to digitize clinical practice guidelines [81], because they lie outside of our inclusion criteria. This complexity is exacerbated in the overall confusion of the PP definitions, as also presented in the Result section of this review.

Identifying the operational definitions of the PP by the authors has been challenging. We filtered articles based on their emphasis on defining and explicitly stating where the focus of their work mainly targets. However, there is a fine line between closely similar concepts, such as workflow and clinical practice guidelines, which were understood as synonyms in a few original papers and previous reviews [12,37,42]. In contrast, even though terms that are used to represent similar concepts with PPs (those that were highlighting the organizational aspects of a care delivery) are presented in the title of the paper, the definition and the emphasis given to the concept in the body of the paper did not demonstrate the intention. These papers were excluded. We did not include patient journeys in the search strategy because it differs from the PP concept definition (in 1 review included synonymously [12]), which resulted in excluding modeling languages that are patient centric, such as CJML.

We have not conducted ontological analysis to check for the correspondence of ontologies and notations [28] or any other quality appraisal frameworks [73]. The use of such tools would have excluded a few papers that are more focused on scripts and are more method-intensive where limited information on the core modeling language and notations were presented. We have not stated a strict definition of a modeling language that implicates the need for a quality appraisal. Identifying the known and standardized modeling languages was simple. Applying strict operational definitions to those lesser-known DSMLs was complex and required careful analysis. Therefore, we considered the authors’ description of the modeling language to accommodate in this scoping review. This resulted in the inclusion of papers focused on techniques and methods with less information about the modeling languages that were used. Some raised important questions about a modeling language but used a more generic description in the part where they described the tool [68].

The variability in extractable data from each paper significantly influenced the focus of our discussion. Despite using the Wand and Weber framework [44], the lack of detailed information within each manuscript constrained our ability to engage in deeper discussions within this review. While the aim of this scoping review was not to conduct quality appraisal, the limitations in available data impeded our ability to thoroughly identify research gaps in a more nuanced manner.

### Gaps Identified for Future Research

Finding effective ways of describing and communicating a PP is essential to enhance its impact. It is as important as having a framework for its development and implementation. A considerable number of frameworks suggesting a standardized development of a PP to set common and shared practices do exist [12,27]. However, less focus has been given on how to simplify and optimize the description and communication of a model beyond the use of customary box-and-arrow type flowcharts and narrative text descriptions. Given the amount of locally made PPs, the effort directed toward the use of visual modeling language is very scarce. A report from PPs in oncology care research group [82] found that many of the cancer care pathways were presented in the form of flowcharts and texts. We also reviewed the national websites where the popular standardized cancer care pathways, for example, locally known as “pakkeforløp for kreft” in Norway, are presented in Scandinavian countries [83-85]. The pathways for several cancer cases were presented in a flowchart, a narrative text, and a table format. We have not explored how they are presented at each institution level, including the process of integration with EHR systems. As per the process steps of the cancer care pathways, the need for a standardized visual modeling language may not be argued provided that the intended goal is achieved by the current representations. This example and the overall limited adoption of visual modeling languages suggest that despite the inherent ambiguity and lack of precision and consistency associated with narrative and text descriptions [62], many institutions still rely on these representations over visual models. This reliance may stem from factors such as organizational culture, lack of awareness, and apprehension about the complexity of modeling languages. However, raising awareness and fostering a better understanding of the benefits and drawbacks of visual models, particularly in the context of specific PP projects, could help shift attitudes and reshape organizational culture regarding the choice of process representation.

Efforts to develop simplified visual modeling languages accessible to nonmodeler domain experts can help alleviate concerns about complexity and encourage broader adoption. While balancing ease of understanding with the expressiveness of modeling languages poses challenges [65], it is essential for all contributions in this field to prioritize this as a principle. In addition, as digital technology plays an increasingly prominent role in this domain, emphasis should be placed on visual languages equipped with execution engines and tools, facilitating integration with existing digital tools, and streamlining the modeling process.

In larger projects intended for widespread implementation, such as those aiming to serve as common documents for local implementations, incorporating visual models of business processes benefits the project while normalizing the use of such representations. For instance, the World Health Organization’s smart guideline documents have integrated visual representations of business processes [86]. However, further research is needed to thoroughly evaluate the advantages and disadvantages of visually modeling such projects using an accessible and expressive modeling language.

Using the decision model to select the most appropriate process-modeling language for a given modeling task is proposed for research modelers, which can be applied in the PP domain as well [74]. Guizani and Ghannouchi [75] argued that none of the languages that they reviewed fully supported the following 7 criteria: expressiveness, flexibility, formality, readability, support tools, usability, and ease of learning. Therefore, multicriteria decision support analysis is suggested as the most appropriate approach for comparison. We recommend that modelers and PP developers should exhaustively scrutinize the suitability of existing standards to model their PP before embarking on developing a DSML from scratch. However, reviews are needed to avail comprehensive knowledge of the gaps in the existing potential candidate languages and weigh the merits of developing new ones in relation to whether all requirements in a PP concept are covered or not.

Extension of existing languages, especially extension by addition, is a good approach to cover domain requirements but the standardization process of the extensions is a challenge [39,49]. By contrast, the latest OMG-recommended combinations of modeling languages have to be tested in different contexts as it is a promising approach to cover all the domain needs [62]. With the move toward a more patient-centric nature of pathways, more research is needed on how to reflect the patients’ views in the modeling product [12,25,87].

### Conclusions

Diverse visual modeling languages were used to model PPs fully or partially. The GPMLs were used directly without any modification to the grammar, extended following extension protocols, or combined with other languages to complement the inherent limitations of each language. We identified a few attempts of developing DSMLs in this review. A limited number of papers presented a DSML that is developed to meet the specific requirements of the PPs. Purely ontological modeling languages were also identified. We have shown the need to consider the rationale, context, and the ways in which the identified visual modeling languages were used. This provides additional useful information to stakeholders in the process of selecting a modeling language. Furthermore, one should use quality appraisal tools to check the conformity of a modeling language to their specific pathway project before deciding to use, extend, combine, or develop a visual modeling language.

## Appendix

Multimedia Appendix 1 Search strategies.

Multimedia Appendix 2 Context in which the modeling languages are applied.

Multimedia Appendix 3 Example diagrams of the customer journey modeling language, adopted from one of the authors’ previous work for illustration purposes; customer journey diagram (upper part): in this case, a patient consulting a general practitioner and being referred to a specialist. The swimlane diagram (lower part) reveals the network of actors involved in the patient’s journey).

Multimedia Appendix 4 PRISMA-ScR (Preferred Reporting Items for Systematic Reviews and Meta-Analyses Extension for Scoping Reviews) checklist.

## Abbreviations

BPM: business process management
BPMN: business process model and notation
BPMN4CP: business process model and notation for care pathways
CJML: customer journey modeling language
CMMN: case management model and notation
DMN: decision model and notation
DSML: domain-specific modeling language
EHR: electronic health record
GPML: general purpose–modeling language
HL7: Health Level Seven International
OMG: Object Management Group
PP: patient pathway
PRISMA-ScR: Preferred Reporting Items for Systematic Reviews and Meta-Analyses Extension for Scoping Reviews
SNOMED-CT: Systematized Nomenclature of Medicine–Clinical Terms
TP: task planning
TP-VML: task planning visual modeling language
UML: unified modeling language
