# Supplementary material for: Visual Modeling Languages in Patient Pathways: Scoping Review
Source: Interact J Med Res. 2024 Nov 15;13:e55865. doi: 10.2196/55865 (PMC11607556; doi:10.2196/55865)
Supplement: Multimedia Appendix 1 [file ijmr_v13i1e55865_app1.docx]

## Multimedia Appendix 1: search strategies

**Documentation on the literature search for: Patient pathways**

The following databases were searched:

| **Database** | **Number of retrieved references** |
| --- | --- |
| MEDLINE (Ovid) | 551 |
| Embase (Ovid) | 741 |
| APA PsycInfo (Ovid) | 432 |
| CINAHL (EBSCO) | 464 |
| Scopus (Elsevier) | 597 |
|  |  |
| Number of records before deduplication: | 2785 |
| Number of duplicate records removed: | 950 |
| Number of records after deduplication: | 1835 |

**Ovid MEDLINE(R) ALL <1946 to November 21, 2022>**

| 1 | Critical Pathways/ or ((patient* or care or clinical or critical) adj (path or paths or pathway* or path-way* or care map*)).ti,kf. | 10070 |
| --- | --- | --- |
| 2 | (conceptual model* or model* language* or process model* or formalism* or meta-model or metamodel or theor* or concept* or framework* or method* or tool* or principle* or definition* or grammar*).ti,kf. | 1131882 |
| 3 | 1 and 2 | 551 |

**Embase Classic+Embase <1947 to 2022 November 21>**

| 1 | clinical pathway/ or ((patient* or care or clinical or critical) adj (path or paths or pathway* or path-way* or care map*)).ti,kf. | 14444 |
| --- | --- | --- |
| 2 | (conceptual model* or model* language* or process model* or formalism* or meta-model or metamodel or theor* or concept* or framework* or method* or tool* or principle* or definition* or grammar*).ti,kf. | 1299645 |
| 3 | 1 and 2 | 741 |

**APA PsycInfo <1987 to November Week 2 2022>**

| 1 | integrated services/ or ((patient* or care or clinical or critical) adj (path or paths or pathway* or path-way* or care map*)).ti,id. | 5434 |
| --- | --- | --- |
| 2 | (conceptual model* or model* language* or process model* or formalism* or meta-model or metamodel or theor* or concept* or framework* or method* or tool* or principle* or definition* or grammar*).ti,id. | 396762 |
| 3 | 1 and 2 | 432 |

**CINAHL (EBSCO)**

| S1 | MH "Critical Path" OR TI ((patient* OR care OR clinical OR critical) N0 (path OR paths OR pathway* OR path-way* OR care map*)) OR SU ((patient* OR care OR clinical OR critical) N0 (path OR paths OR pathway* OR path-way* OR care map*)) | 7,806 |
| --- | --- | --- |
| S2 | TI ("conceptual model*" OR "model* language*" OR "process model*" OR formalism* OR meta-model OR metamodel OR theor* OR concept* OR framework* OR method* OR tool* OR principle* OR definition* OR grammar*) | 204,485 |
| S3 | S1 AND S2 | 464 |

**Scopus**

TITLE ( ( ( patient* OR care OR clinical OR critical ) W/0 ( path OR paths OR pathway* OR path-way* OR "care map*" ) ) AND ( "conceptual model*" OR "model language*" OR "process model*" OR formalism* OR meta-model OR metamodel OR theor* OR concept* OR framework* OR method* OR tool* OR principle* OR definition* OR grammar* ) )

597 hits.
