# Supplementary material for: Visual Modeling Languages in Patient Pathways: Scoping Review
Source: Interact J Med Res. 2024 Nov 15;13:e55865. doi: 10.2196/55865 (PMC11607556; doi:10.2196/55865)
Supplement: Multimedia Appendix 2 [file ijmr_v13i1e55865_app2.doc]

## Multimedia Appendix 2: Context in which the modeling language(s) applied

| **Author, year** | **Modeling language** | | **Context of the modeling application** | |
| --- | --- | --- | --- | --- |
| **Braun et al.,** [1]**, 2016** | BPMN4CP | | Provides detailed methodology for extending BPMN to healthcare domain  Follows and enhances the extension mechanisms, including the prior version  Used as a base for extending the specification to health domain, and patient pathway concept | |
| **Richter and Schlieter** [2]**, 2019** | BPMN, with Quality Indicator extension | | Requirement analysis conducted on the original specification.  BPMN4CP used as a starting extension to the domain  Quality indicator extension added following the extension standard  Domain and modeling experts participated  Evaluated extension with domain experts | |
| **Tehrani et al.,** [3]**, 2013** | BPMN, with Norm extension | | Organizational semiotics method with norm-analysis  The results of the norm analysis added as an “extension” labelling [N#] to the BPMN formalism  No indication of following BPMN extension guidance  Demonstrated on major gynecological surgery (adding pressure ulcer classification and management)  Positive usability results from clinicians | |
| **Iglesias et al.,** [4]**, 2022** | Task Planning -Visual Modeling Language [TP-VML]) | | Part of *openEHR Task Planning* (TP) specifications; clinical oriented formalism  Method included analysis of domain requirements and BPMN extension, features of TP formalisms, case-study (Catheter-related bloodstream infection  ), and the proposal for extension  TP-VML has a formal semantics, supports automatic translation of graphical representation to executable models  Nondeterministic/handles dynamicity  Able to represent the same logic in extended BPMN using TP-VML | |
| **Trajano et al.,** [5]**, 2021** | MedPath | | Model-Based Engineering  Simplified presentation to the level of boxes-and-arrow  Programmable and automated execution of pathways into interactive systems  For both expert and non- expert modelers  Usability test: positive  Users were satisfied | |
| **Burwitz et al.,** [6]**, 2013** | CP-Mod | | Adaption and development of extension  Used Clinical Algorithm, a modeling of clinical practice guideline and extended to accommodate all the requirements of PP  Discusses all levels of abstraction; applied on IT system –low level  Striving for both human and machine-readable formalisms | |
| **Shitkova et al.,** [7]**, 2015** | Icebricks | | Application study  Using a web-based process modeling application, a comprehensive methodology and modeling tool developed  Modeling can be done at four layers of abstractions; graphical representation of processes in an organization to ‘process bricks’  Originally developed in other sector before adapted to clinical pathways (only changes were the customization of glossary and attribute groups). | |
| **Combi et al.,** [8]**, 2017** | | BPMN & DMN | | Application; combination  A framework around the implementation and combination of two well-known modeling languages  Produced an XML version that can be read by MS-office |
| **Sooter et al.,** [9]**, 2019** | | BPMN & DMN | | Application; combination  Modeling the recommendation of medical eligibility criteria for contraceptive initiation and continuation; incorporating into medical health record  Work-around required in the application process –a ‘spreadsheet’  Process reveals extension recommendation to the BPMN2.0 specification  Models and software designers  Low level of abstraction |
| **OMG,** [10]**, 2019** | | BPM+ (BPMN, CMMN & DMN) | | Guidance; combination  A guideline comprehensive methodology and techniques presented  Uptake by HIT/ integration to EHR as the guideline supports the execution integrating other specifications  To all ‘modeling team’ including those without expertise in modeling  Not endorsed as a standard; a discussion paper  (Respective grammars, methods, notations and scripts linked to the OMG website)  Level of abstraction depends on the goal of adoption |
| **Bowles et al.,** [11]**, 2018** | | BPMN & LES (Labelled event structure) | | Combination of modeling languages and techniques  Focuses on automated detection of comorbidity pathway conflicts and propose the best pathway.  The application produced at the end, in a way to present the conflicts analyzed by the back-end system that reads from the front-end and presented in a graphical format (the authors stated that it is primitive) to the end users.  Low level of abstraction, with intense machine execution using proprietary software |
| **Ardito et al.,** [12]**, 2020** | | A meta-model; based on an Enhanced entity relationship (EER) data model with a simplified BPMN and UML activity diagram | | Application study: method intensive  A meta-model that allows execution of pathway knowledge using general purpose description language formalisms independently from graphical representation, that can adopt any standard. The approach bridges the complex requirements for automation and human understandable representation via social media integrated chatbot in a simple natural language  Engaging patient in the execution of clinical pathways  The process is modeled and Task-oriented chatbot works following modeled clinical pathways  The meta-model:  Step 1: defining the domain by means of EER model  Step 2; representing the EER data model in terms  of a UML class diagram |

Reference

1. Braun R, Schlieter H, Burwitz M, Esswein W. BPMN4CP Revised - Extending BPMN for Multiperspective Modeling of Clinical Pathways. 2016. doi: 10.1109/HICSS.2016.407

2. Richter P, Schlieter H. Process-Based Quality Management in Care: Adding a Quality Perspective to Pathway Modelling. In: Panetto H, Debruyne C, Hepp M, Lewis D, Ardagna CA, Meersman R, editors. Move Meaningful Internet Syst OTM 2019 Conf Cham: Springer International Publishing; 2019. p. 385–403. doi: 10.1007/978-3-030-33246-4_25

3. Tehrani J, Liu K, Michell V. Semiotics-oriented method for generation of clinical pathways. 2013. p. 477–482. doi: 10.1007/978-3-642-32054-5_69

4. Natalia I, Jose M J, Manuel C. Business Process Model and Notation and openEHR Task Planning for Clinical Pathway Standards in Infections: Critical Analysis. J Med Internet Res J Med Internet Res; 2022 Sep 15;24(9). PMID:36107480

5. Trajano IA, Ferreira Filho JB, de Carvalho Sousa FR, Litchfield I, Weber P. MedPath: A process-based modeling language for designing care pathways. Int J Med Inf 2021;146:104328. doi: 10.1016/j.ijmedinf.2020.104328

6. Burwitz M, Schlieter H, Esswein W. Modeling Clinical Pathways - Design and Application of a Domain-Specific Modeling Language. Wirtsch Proc 2013 2013 Jan 1; Available from: https://aisel.aisnet.org/wi2013/83

7. Shitkova M, Taratukhin V, Becker J. Towards a methodology and a tool for modeling clinical pathways. 2015. p. 205–212. doi: 10.1016/j.procs.2015.08.335

8. Combi C, Oliboni B, Zardini A, Zerbato F. A Methodological Framework for the Integrated Design of Decision-Intensive Care Pathways-an Application to the Management of COPD Patients. J Healthc Inform Res 2017;1(2):157–217. doi: 10.1007/s41666-017-0007-4

9. Sooter LJ, Hasley S, Lario R, Rubin KS, Hasić F. Modeling a Clinical Pathway for Contraception. Appl Clin Inform 2019 Oct;10(5):935–943. PMID:31860113

10. Object Management Group. Field Guide to Shareable Clinical Pathways, v.2.0 Discussion Paper. OMG; Available from: https://go.omgprograms.org/l/658223/2019-05-17/41hdg [accessed Oct 12, 2023]

11. Bowles J, Caminati MB, Cha S. An integrated framework for verifying multiple care pathways. 2018. p. 1–8. doi: 10.1109/TASE.2017.8285628

12. Ardito C, Caivano D, Colizzi L, Dimauro G, Verardi L. Design and execution of integrated clinical pathway: A simplified meta-model and associated methodology. Inf Switz 2020;11(7). doi: 10.3390/info11070362
