# Supplementary figures and images for: Visual Modeling Languages in Patient Pathways: Scoping Review
Source: Interact J Med Res. 2024 Nov 15;13:e55865. doi: 10.2196/55865 (PMC11607556; doi:10.2196/55865)

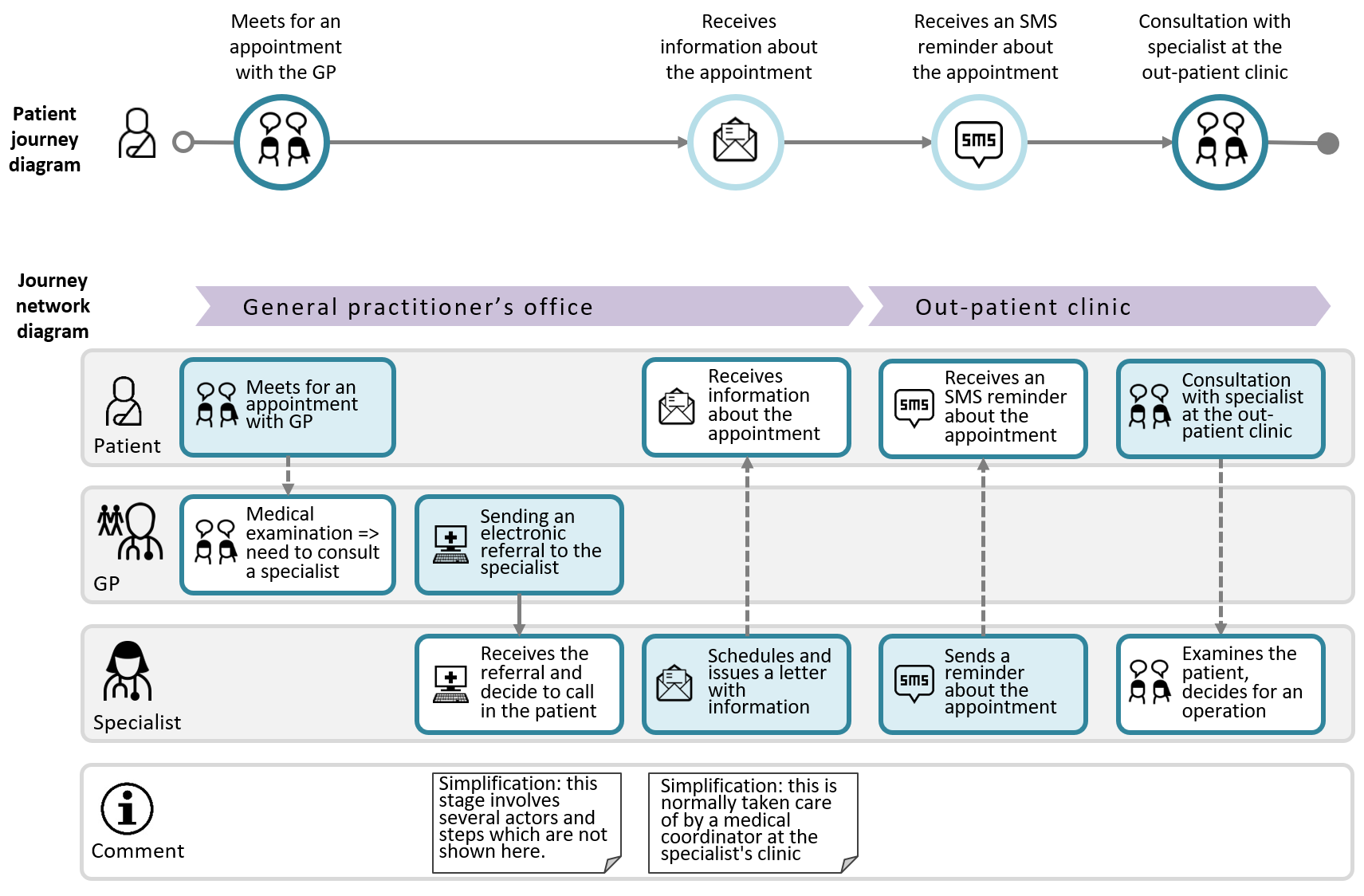

Supplement: Multimedia Appendix 3 [file ijmr_v13i1e55865_app3.png]
